# Sex‐dependent effects of parental age on offspring fitness in a cooperatively breeding bird

**DOI:** 10.1002/evl3.300

**Published:** 2022-11-16

**Authors:** Alexandra M. Sparks, Martijn Hammers, Jan Komdeur, Terry Burke, David S. Richardson, Hannah L. Dugdale

**Affiliations:** ^1^ Faculty of Biological Sciences, School of Biology University of Leeds Leeds LS2 9JT United Kingdom; ^2^ School of Biosciences University of Sheffield Sheffield S10 2TN United Kingdom; ^3^ Groningen Institute for Evolutionary Life Sciences University of Groningen Groningen 9712 CP The Netherlands; ^4^ Aeres University of Applied Sciences Almere 1325 WB The Netherlands; ^5^ School of Biological Sciences University of East Anglia Norwich NR4 7TJ United Kingdom; ^6^ Nature Seychelles Mahé Republic of Seychelles

**Keywords:** Ageing, fitness, intergenerational effects, Lansing effect, life span, maternal age effect, paternal age effect, senescence, Seychelles warbler

## Abstract

Parental age can have considerable effects on offspring phenotypes and health. However, intergenerational effects may also have longer term effects on offspring fitness. Few studies have investigated parental age effects on offspring fitness in natural populations while also testing for sex‐ and environment‐specific effects. Further, longitudinal parental age effects may be masked by population‐level processes such as the selective disappearance of poor‐quality individuals. Here, we used multigenerational data collected on individually marked Seychelles warblers (*Acrocephalus sechellensis*) to investigate the impact of maternal and paternal age on offspring life span and lifetime reproductive success. We found negative effects of maternal age on female offspring life span and lifetime reproductive success, which were driven by within‐mother effects. There was no difference in annual reproductive output of females born to older versus younger mothers, suggesting that the differences in offspring lifetime reproductive success were driven by effects on offspring life span. In contrast, there was no association between paternal age and female offspring life span or either maternal or paternal age and male offspring life span. Lifetime reproductive success, but not annual reproductive success, of male offspring increased with maternal age, but this was driven by between‐mother effects. No paternal age effects were found on female offspring lifetime reproductive success but there was a positive between‐father effect on male offspring lifetime reproductive success. We did not find strong evidence for environment‐dependent parental age effects. Our study provides evidence for parental age effects on the lifetime fitness of offspring and shows that such effects can be sex dependent. These results add to the growing literature indicating the importance of intergenerational effects on long‐term offspring performance and highlight that these effects can be an important driver of variation in longevity and fitness in the wild.

**Impact summary**

In virtually all animals, an individual's health and condition deteriorate with age (senescence), which impacts their survival and the number of offspring produced in later life. Importantly, the quality of offspring produced, as measured through their physiological condition, survival, and reproductive success, may also be impacted by their parents’ ageing. This may have considerable health and evolutionary implications. Most of the evidence for intergenerational effects of parental age comes from studies on laboratory animals that are kept under artificial conditions. However, little is known about such effects in natural populations, where a myriad of different stresses and strains act upon individuals. Furthermore, it remains unclear whether parental age effects on offspring fitness are sex specific or affected by environmental conditions. Our study reveals that, in a wild‐living bird, parental age effects on offspring life span and lifetime fitness depend on both the sex of the parent and the sex of the offspring. Cross‐sectionally, we found negative effects of maternal age, but no effects of paternal age, on the life span and lifetime reproductive success of female offspring. In contrast, although there were no parental age effects on male offspring life span, there was a positive between‐mother and between‐father effect on male offspring lifetime reproductive success. Our study highlights that not including such intergenerational age effects, not separating within‐ and between‐parental effects, and not testing for sex‐specific effects may underestimate or obscure important components of ageing and senescence in wild populations. These intergenerational effects could have important implications for population dynamics, particularly in age‐structured populations, for example, in terms of conservation management and evolutionary dynamics.

Early‐life environmental conditions can have considerable consequences for later‐life individual fitness. Parental effects, whereby an offspring's phenotype is influenced by their mother's or father's phenotype, above and beyond the genes they inherit, are widespread in natural populations (Mousseau and Fox 1998) and can have important consequences for ecological and evolutionary dynamics (Kirkpatrick and Lande 1989). Although the impact of parental effects is typically largest in early life (Moore et al. 2019), a growing number of studies have indicated that parental effects can have long‐term effects on offspring survival and fitness (Arslan et al. 2017; Bock et al. 2019).

Due to the deterioration of individuals with age in a process known as senescence, parental age is an important component of parental effects with the potential to have considerable negative consequences on offspring phenotype and performance. For instance, a negative effect of parental age on offspring life span has been reported across a range of taxa in both laboratory and field studies (“Lansing effect”; Monaghan et al. 2020). Age‐dependent declines in parental gamete quality (Monaghan and Metcalfe 2019) and declines in parental care (Hammers et al. 2021) are predicted to cause reduced fitness in offspring of elderly parents. Indeed, there is increasing evidence in wild mammal and bird populations that elderly parents produce offspring with lower birth weights and reduced neonatal survival (e.g., Hoffman et al. 2010; Hayward et al. 2015; Fay et al. 2016). However, due to the necessity for long‐term longitudinal studies with accurate lifetime reproductive success (LRS) and survival data, the number of studies that have investigated the impact of parental age on the life span and LRS of offspring in natural populations remains limited to a few wild mammal (European rabbits [*Oryctolagus cuniculus*], Asian elephants [*Elephas maximus*], yellow‐bellied marmots [*Marmota flaviventer*]), and bird (great tits [*Parus major*], common terns [*Sterna hirundo*], house sparrows [*Passer domesticus*]) populations (Rödel et al. 2009; Bouwhuis et al. 2010, 2015; Schroeder et al. 2015; Reichert et al. 2019; Kroeger et al. 2020). Results from these studies have been mixed. Further, longitudinal parental age effects may be obscured by population‐level processes such as the selective (dis)appearance of poor‐quality individuals, but these can be separated into within‐ and between‐parental age effects using within‐subject centering (van de Pol and Wright 2009). However, of these studies in natural populations, only two have separated within‐ from between‐parental age effects to account for the effects of selective (dis)appearance (Bouwhuis et al. 2015; Reichert et al. 2019) and only two have investigated both maternal and paternal age effects on offspring of both sexes (Bouwhuis et al. 2015; Schroeder et al. 2015).

Parental age studies typically focus on maternal age effects, because maternal investment is usually higher than paternal investment and, depending on the study system, data on females are typically easier to obtain. However, an increasing number of studies are documenting paternal age effects on offspring performance (e.g., Fay et al. 2016). In addition, offspring sex‐dependent effects of parental age have been observed (Bouwhuis et al. 2015; Schroeder et al. 2015). These effects may be due to differences between the sexes in their responses to their early‐life environment or sex‐specific epigenetic inheritance or investment (Bouwhuis et al. 2015). Further, although early‐life environmental conditions have been shown to affect offspring fitness (Monaghan 2008), only one study has investigated how such conditions may interact with parental age (Kroeger et al. 2020). More longitudinal studies investigating both maternal and paternal age effects and testing whether these effects are dependent on offspring sex, or the environment, are needed to fully assess the importance of parental age effects.

Here, we examine the relationships between parental age and offspring life span and LRS using the long‐term study on the Seychelles warbler (*Acrocephalus sechellensis*) population on Cousin Island. This facultatively cooperative‐breeding bird lives in and defends territories in which a dominant male and female reside. Around half of these territories may also contain subordinate individuals, of which approximately one third help with parental care in any given year (Hammers et al. 2019). A combination of high annual resighting rates (Brouwer et al. 2010), virtually no interisland dispersal (Komdeur et al. 2004), and low extrinsic mortality means that the birds live long lives (up to 19 years) and accurate hatch and death years can be estimated (Hammers et al. 2015). High levels of extra‐pair paternity and subordinate female breeding occur (Raj Pant et al. 2019), but a genetically verified pedigree allows accurate estimation of LRS (Sparks et al. 2021). Furthermore, the high extra‐pair paternity allows us to separate the effects of the age of the genetic father (e.g., germline deterioration) from that of the social father (deterioration in parental care).

Actuarial and reproductive senescence have been well documented in the Seychelles warbler (Hammers et al. 2012, 2013; Raj Pant et al. 2020). Mothers provide more parental care to the offspring than fathers (Hammers et al. 2019) and the amount of provisioning declines with maternal but not paternal age (Hammers et al. 2021). Further, the first‐year survival of offspring declines with maternal but not paternal age (Hammers et al. 2021). The presence of helpers, however, does compensate for age‐related declines in parental care and offspring first‐year survival with dominant female age (Hammers et al. 2021), providing a means by which social conditions in early life could influence offspring fitness. The long‐term effects of parental age on offspring life span and LRS remain unknown.

In this study, we use a 24‐year dataset on individually marked Seychelles warblers to investigate whether maternal and paternal ages are associated with the life span and LRS of offspring. We investigate whether offspring fitness is associated with the age of the mother, genetic father, and social father. We also investigate whether parental age effects are dependent on the offspring's sex or early‐life environment. We predict that beneficial early‐life environments may counteract the negative impacts of parental age effects on offspring fitness, whereas negative early‐life environments may amplify these effects.

## Methods

### STUDY SYSTEM

The Seychelles warbler is a small insectivorous passerine bird endemic to the Seychelles archipelago. Since 1985, the entire population (about 320 adult individuals in 115 territories) on Cousin Island (04′20′S, 55′40′E) has been monitored intensively, although a small number of birds on the island were ringed before the project began (Komdeur 1991; Hammers et al. 2015). The dominant breeding pair in each territory is determined by behavioral observations of contact calls and mate guarding (Richardson et al. 2002). The main breeding season runs from June to September and coincides with the peak of insect abundance (Komdeur et al. 1991). However, a smaller proportion of individuals also breed between January and March. On Cousin, most clutches contain one egg, although clutches of up to three eggs can occur (Richardson et al. 2001). Although one female can lay more than one egg, the presence of multiple eggs in a nest is often indicative of subordinate females breeding (Richardson et al. 2002). Subordinate female co‐breeding contributes to 11% of maternities (Raj Pant et al. 2019), and it is always by subordinate females within the territory, with no evidence of egg dumping by females from other territories (Richardson et al. 2001). Extra‐group paternity accounts for 41% of paternities (Raj Pant et al. 2019). Only 0.6% of paternities are gained by (within‐group or extra‐group) subordinate males (Sparks et al. 2021).

Fieldwork is carried out during the breeding seasons, when as many birds as possible are caught using mist nests or captured in the nest. The age of unringed birds is estimated based on lay, hatch, or fledge dates and/or using eye color (Komdeur et al. 1991). Since 1995, blood samples have been collected by brachial venipuncture from all birds captured and stored at room temperature in 1 mL of absolute ethanol for molecular sexing and genetic pedigree construction. From 1997 onward, >96% of individuals in the population have been individually marked with a unique combination of a British Trust for Ornithology metal ring and three color rings (Brouwer et al. 2010).

To investigate the influence that early‐life conditions have on parental age effects, we investigated the effect of natal territory quality, group size, and helper presence in the offspring's natal territory. We used an index of insect abundance as a measure of territory quality as warblers are almost entirely insectivorous (Komdeur and Daan 2005). Territory quality was calculated as previously described (van de Crommenacker et al. 2011; Brouwer et al. 2012; see *Methods* in the Supporting Information for full details).

We used natal group size and helper presence as proxies for the early‐life social environment. Natal group size was the number of independent individuals (i.e., two breeders and any subordinates) in the natal territory (range: 2−6). Helpers are subordinates observed to help incubate or provision offspring in a specific breeding season (0−3 per territory). Due to few instances of >1 helper in the dataset (*N* = 43 of 1321), helper presence in the natal territory was included as a binary variable (present/absent).

### STATISTICAL ANALYSES

Our dataset included all individuals that were hatched 1995−2018 with assigned parentage who survived to at least 1 year of age (1321 offspring from 437 mothers and 417 fathers hatched between 1986 and 2016). We investigated parental age effects with genetic parents identified using MasterBayes version 2.5.2 (Hadfield et al. 2006) following Sparks et al. (2021). The Seychelles warbler pedigree includes parentage assignments for individuals hatched between 1992 and 2018, but data were most complete from 1995. Parentage was assigned where *P* ≥ 0.8. Although 11% of maternities are from subordinate females, there is no difference in the provisioning rates of subordinate or dominant females at nests where a subordinate offspring is present (Table S1). Hence, in this dataset we take the genetic mother as the social mother. In addition to the genetic father, we also included the age of the dominant male in models (59% of offspring are sired by the dominant male; Raj Pant et al. 2019). However, the conclusions were the same if models were run separately with just one of the two paternal ages (Table S2). All statistical analyses were performed in R version 3.6.3 (R Core Team 2019) and all models were run separately for male and female offspring to allow for differences in LRS and life span between the sexes (Fig. S1) and to improve interpretability of interactions.

Life span was calculated for all individuals who survived to at least 1 year of age. Because the resighting probability is high, an individual's death year was assigned as the last sighting year. Life span analyses were performed using Poisson generalized linear mixed models (GLMM) in glmmTMB version 1.0.2.1 (Brooks et al. 2017) for all individuals who were not translocated as part of a conservation program (Wright et al. 2014) and whose last seen year was before 2019. We included the ages of the genetic mother, genetic father, and dominant male as fixed effects (linear and squared covariates), in addition to hatch year (linear covariate), the natal group size (linear covariate), presence of helpers in the natal territory (binary factor), natal territory quality (linear covariate), and the presence of siblings in the nest (binary factor). Offspring hatch year was included to account for the fact that individuals hatched more recently in the dataset lived short lives (following Bouwhuis et al. 2015). Hatch year and maternal, paternal, and dominant male identity were included as random effects. To calculate a hazards ratio for parental age on offspring survival, we also ran these models as a Cox proportional hazards mixed‐effects model in package coxme version 2.2‐16 (Therneau 2019) using the same model structure but without hatch year as a covariate as some individuals in these models were still alive. In these models, individuals (129 females, 141 males) that were still alive (i.e., observed in 2019 or 2020) or translocated to other islands (31 females, 44 males) were right censored. In both models, we subsequently tested for environment‐dependent parental age effects by testing for two‐way interactions of natal territory quality, group size, and helper presence with linear ages of the mother, father, and dominant male. We further tested for maternal × paternal age interactions on offspring life span. Finally, we tested whether any parental age effects on life span translated to differences in reproductive life span (see *Methods* in the Supporting Information for full details).

LRS, calculated as the sum of offspring produced who survived to at least 1 year of age, was calculated for all individuals who survived to at least 1 year of age, were not translocated, and whose last seen year was before 2018 (last year of the pedigree). Analyses were performed using a zero‐inflated Poisson GLMM in glmmTMB. It included the same fixed and random effects as the life span models. As for the life span models, we subsequently tested for maternal × paternal age interactions and environment‐dependent parental age effects by including interactions between parental age and natal group size, helper presence, and natal territory quality. To test whether any effects of parental age may be mediated by offspring life span, we included offspring life span in our models. Alternatively, parental age effects on offspring LRS may be mediated by differences in annual reproductive output of offspring. To test this, we also ran models of annual reproductive success (see *Methods* in the Supporting Information for full details).

Any parental age effects observed at the cross‐sectional level may not reflect age‐specific variation within parents because both within‐ and between‐parental effects can drive patterns at the population level. In wild populations, there is often variation between individuals in the onset of breeding as well as life span. Such variation can mask parental ageing effects, or could generate spurious results if a parent's life span rather than ageing per se is related to offspring life span and LRS (e.g., Reid et al. 2010). Consequently, we investigated whether any parental age effects on offspring life span and LRS were driven by within‐parent age effects (age‐specific variation in offspring life span and LRS within individual parents) or between‐parent age effects (e.g., selective (dis)appearance of poor‐quality parents). To do this, we used the within‐subject centering method (van de Pol and Wright 2009; see *Methods* in the Supporting Information for full details). This method centers all measurements of the parent around their mean age, thus eliminating between‐subject variation. Between‐parent effects could be caused by anything driving differences in a parent's mean age. For example, positive between‐parent effects could be caused by parents surviving to or starting to breed at older ages having offspring with higher fitness. After accounting for between‐parent effects, the within‐parent effects subsequently reflect true age‐specific variation in offspring life span and LRS within individual parents.

Correlations between age of the mother and the age of the genetic and/or social father were weak, which allowed them to be included in the same model (maternal age and paternal age: Pearson's correlation; *r* = 0.085, *t*_1319_ = 3.109, *P* = 0.002; Fig. S2; maternal age and dominant male age: Pearson's correlation; *r* = 0.070, *t*_1299_ = 2.538, *P* = 0.011). The correlation between the age of the genetic father and dominant male was moderate (Pearson's correlation; *r* = 0.557, *t*_1299_ = 24.187, *P* < 0.001), which was unsurprising given that 59% of offspring are sired by the dominant male within the territory (Raj Pant et al. 2019). We checked for collinearity between fixed effects by calculating variance inflation factors (all <2), as well as overdispersion in all models. To help with model convergence and aid interpretation of the model coefficients, continuous predictor variables were standardized by subtracting the mean and dividing by two standard deviations in arm version 1.11‐2 (Gelman and Su 2018). Significance of fixed effects was determined by likelihood ratio tests comparing a model with and without the fixed effect of interest. Nonsignificant (i.e., *P* > 0.05) age^2^ effects and interactions were removed from the models sequentially, in order of least significance, to ensure their inclusion did not affect interpretation of the first‐order effects. All dropped terms were retested against the base model using likelihood ratio tests.

## Results

### PARENTAL AGE AND OFFSPRING LIFE SPAN

**Figure 1:**
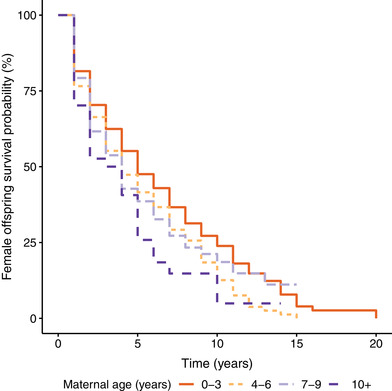
Survival probability of female Seychelles warbler offspring over time broken down by age of the mother in the cross‐sectional analyses. Dataset includes all females who survived to at least 1 year who were included in the Cox proportional hazards mixed‐effect model in Table S3 (*n* = 627). Maternal age was treated as a continuous variable in our analyses, but for graphical purposes, here maternal age is grouped into four age classes: 0–3 years, 4–6 years, 7–9 years, and 10+ years.

There was a sex‐dependent association between maternal age and offspring life span. Older mothers produced female offspring with shorter life spans (Tables 1 and S3; Fig. 1). This effect translated into a 3% increase in mortality risk for female offspring for each year that the mother was older (Table S3) and appeared to be driven by a negative within‐mother age effect. As individual mothers aged, the female offspring they produced had shorter life spans (Tables S4 and S5; Fig. S3; see *Results* in the Supporting Information). There was no significant association between maternal age and life span for male offspring (Tables 1 and S3). Further, there was no effect of paternal age on offspring life span of either sex (Tables 1 and S3). There was no support for quadratic parental age effects on offspring life span, or maternal × paternal age interactions (Tables S6 and S7). Similar results were found for offspring reproductive life spans; older mothers produced female offspring with shorter reproductive life spans, but maternal age had no impact on the reproductive life spans of male offspring (Table S8). Paternal age had no effect on the reproductive life spans of offspring of either sex (Table S8).

**Table 1:** Generalized linear mixed model results investigating associations between parental age and offspring life span, for male and female offspring separately, in the Seychelles warbler (including all genetically assigned offspring who survived to at least 1 year)

	Female Offspring Life Span				Male Offspring Life Span			
	*n* = 467				*n* = 485			
Variables	Estimate	SE	LRT	*P*	Estimate	SE	LRT	*P*
Fixed effects								
Intercept	1.276	0.061			1.196	0.072		
Age of mother	**–0.242**	**0.075**	**10.569**	**0.001**	0.098	0.068	2.098	0.148
Age of father	–0.041	0.088	0.217	0.641	–0.062	0.085	0.545	0.460
Age of dominant male	0.135	0.082	2.676	0.102	0.125	0.081	2.363	0.124
Hatch year	**–0.584**	**0.108**	**22.852**	**<0.001**	**–0.556**	**0.138**	**13.710**	**<0.001**
Territory quality	0.019	0.081	0.054	0.816	0.149	0.093	2.598	0.107
Group size	–0.109	0.077	1.994	0.158	–0.075	0.080	0.863	0.353
Helper presence (*y*)	–0.153	0.092	2.787	0.095	–0.128	0.101	1.631	0.202
Sibling presence (*y*)	–0.068	0.103	0.439	0.508	0.135	0.100	1.835	0.176
Random effects								
Maternal ID	0.085				0.086			
Paternal ID	0.163				0.084			
Dominant male ID	0.019				0.068			
Hatch year	0.021				0.068			

*Note*: Included are the parameter estimates (estimate), their standard errors (SE), and the significance of fixed effects based on a likelihood ratio test (LRT, P) where d.f. = 1. Significance of dropped fixed effects (squared parental ages, maternal × paternal age interactions, and environment × parental age interactions) is shown in Table S6. Significant fixed effects are in bold.

We found no interactions between parental ages and early‐life conditions on offspring life span. This suggests there was no evidence for environment‐dependent parental effects (Tables S6 and S7).

### PARENTAL AGE AND OFFSPRING LRS

**Figure 2:**
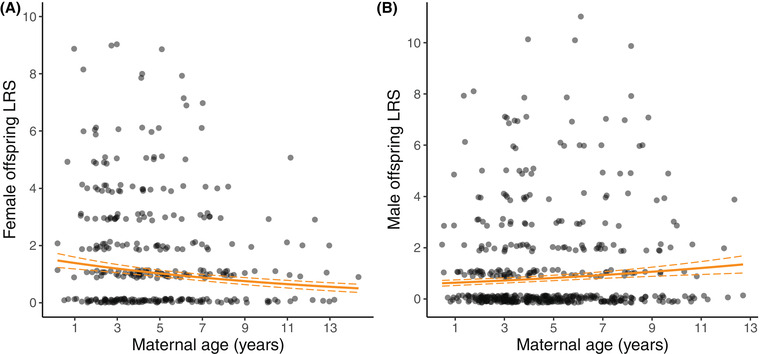
Cross‐sectional maternal age effects on the LRS of female (A) and male (B) offspring (based on genetically assigned offspring who survived to at least 1 year) in the Seychelles warbler. Solid lines indicate GLMM predictions with mean values for all other continuous fixed effects in the model and dashed lines indicate standard errors (Table 2). Dots show the raw data points (A: *n* = 441; B: *n* = 466). LRS values are integers but are jittered to show overlapping values.

Cross‐sectionally, females with older mothers at hatching had lower LRS (Table 2; Fig. 2A). This was driven by a within‐mother age effect: mothers produced female offspring with lower LRS as they aged (Tables S9 and 10; Fig. S4; see *Results* in the Supporting Information). In contrast, there was a weaker but positive association between maternal age and the LRS of male offspring (Table 2; Fig. 2B) driven by a positive between‐mother age effect (Tables S9 and S10; Fig. S4). There was no cross‐sectional effect of paternal age, or age of the dominant male, on the LRS of offspring of either sex (Table 2). However, there was a positive between‐father age effect on male offspring LRS (Tables S9 and S10; Fig. S4). Cross‐sectionally, mothers and fathers that survived to, or started to breed at, older ages had male offspring with higher LRS (Tables S9 and S10; Fig. S4). There was no evidence for quadratic parental age effects on offspring LRS, or maternal × paternal age interactions (Table S11).

**Table 2:** Generalized linear mixed model results investigating associations between parental age and offspring LRS for male and female offspring separately in the Seychelles warbler (including all genetically assigned offspring who survived to at least 1 year)

	Female Offspring LRS				Male Offspring LRS			
	*n* = 441				*n* = 466			
Variables	Estimate	SE	LRT	*P*	Estimate	SE	LRT	*P*
Fixed effects								
Intercept	0.506	0.128			0.298	0.143		
Age of mother	**–0.427**	**0.152**	**7.817**	**0.005**	**0.323**	**0.140**	**5.297**	**0.021**
Age of father	0.145	0.162	0.798	0.372	0.265	0.174	2.295	0.130
Age of dominant male	–0.083	0.158	0.276	0.599	0.023	0.154	0.023	0.879
Hatch year	**–1.082**	**0.221**	**25.568**	**<0.001**	**–1.169**	**0.273**	**17.446**	**<0.001**
Territory quality	–0.135	0.143	0.892	0.345	0.245	0.199	1.495	0.222
Group size	–0.032	0.151	0.046	0.830	–0.144	0.172	0.708	0.400
Helper presence (*y*)	**–0.635**	**0.203**	**10.055**	**0.002**	–0.220	0.215	1.069	0.301
Sibling presence (*y*)	–0.078	0.191	0.167	0.683	0.081	0.205	0.156	0.693
Random effects								
Maternal ID	0.134				0.155			
Paternal ID	0.104				0.265			
Dominant male ID	0.030				<0.001			
Hatch year	0.050				0.105			

*Note*: Included are the parameter estimates (estimate), their standard errors (SE), and the significance of fixed effects based on a likelihood ratio test (LRT, P) where d.f. = 1. Significance of dropped fixed effects (squared parental ages, maternal × paternal age interactions, and environment × parental age interactions) is shown in Table S11. Significant fixed effects are in bold.

There was a strong positive correlation between life span and LRS (Pearson's correlation; *r* = 0.780, *t*_923_ = 37.825, *P* < 0.001; Fig. S5). When we added longevity as a covariate to the model, the association between maternal age and female offspring LRS was no longer significant (maternal age *β* = 0.031 ± 0.131 SE, *χ* ^2^_(1)_ = 0.057, *P* = 0.812; life span *β* = 1.743 ± 0.117 SE, *χ* ^2^_(1)_ = 204.930, *P* < 0.001), which suggests that differences in LRS between female offspring born to different age mothers were driven by differences in longevity rather than by differences in annual reproductive output. In support of this, we found no association between parental age and the annual reproductive success of either female or male offspring (Table S12).

There was no strong evidence for environment‐dependent parental age effects on offspring LRS (Table S11). There was a marginally significant paternal age × territory quality and dominant male age × territory quality interaction on female offspring LRS (Table S11). It appeared that older males in higher quality territories produced female offspring with lower LRS than older males in lower quality territories (Figs. S6–S9). However, this interaction was driven by a few outliers and the errors around these effects were large (Figs. S6–S9).

## Discussion

We found evidence for sex‐dependent parental age effects on offspring fitness in the Seychelles warbler, with negative within‐mother age effects for female offspring but positive between‐mother age effects for male offspring (summarized in Table 3). Female offspring born to older mothers had shorter life spans and lower LRS than females born to younger mothers, which appeared to be driven by within‐mother age effects. Male offspring born to older mothers had higher LRS than males born to younger mothers, which appeared to be driven by between‐mother age effects. Although there were no cross‐sectional paternal age effects, there was a positive between‐paternal age effect of the genetic father on male offspring LRS.

**Table 3:** Summary of the models investigating parental age effects on offspring life span and LRS in the Seychelles warbler

		Age of Mother				Age of Genetic Father				Age of Dominant Male			
		Cross‐sectional	w/n	b/n	Diff.	Cross‐sectional	w/n	b/n	Diff.	Cross‐sectional	w/n	b/n	Diff.
Offspring life span	Females	**–ve**	**–ve**	ns	**Y**	ns	ns	ns	N	ns	ns	ns	N
	Males	ns	ns	ns	N	ns	ns	ns	N	ns	ns	ns	N
Offspring LRS	Females	**–ve**	**–ve**	ns	N	ns	ns	ns	N	ns	ns	ns	N
	Males	**+ve**	ns	**+ve**	N	ns	ns	**+ve**	N	ns	ns	ns	N

*Note*: Included are the cross‐sectional effects, within‐parent (w/n) and between‐parent (b/n) age effects, and whether there was a significant difference between the within‐ and between‐parent age slopes (Diff.). Positive estimates are indicated by +ve, negative estimates are indicated by ‐ve, ns indicates nonsignificant results, and Y and N indicate yes and no, respectively. Significant effects are highlighted in bold.

Although the exact mechanisms are unclear, direct effects of ageing parents on offspring phenotypes could be caused by age‐dependent germline deterioration (Monaghan and Metcalfe 2019), declines in the quality of parental care (Hammers et al. 2021), or age‐dependent increases in the costs of inbreeding depression (Charlesworth and Hughes 1996). The high extra‐pair paternity in the Seychelles warbler allowed us to compare the effects of age of the genetic father (e.g., germline deterioration and epigenetic inheritance) from that of the social father (e.g., deterioration in parental care). However, we only found within‐parent age effects of the mother on offspring. The greater impact of maternal age on offspring performance is perhaps unsurprising given that only females incubate and females provision more than males (Hammers et al. 2019; van Boheemen et al. 2019). Further, age‐dependent declines in provisioning rates occur for dominant females but not males, which also suggests that maternal rather than paternal age should be more important for offspring quality in this population (Hammers et al. 2021).

Although negative effects of maternal age on early‐life offspring survival have been widely reported (e.g., Hoffman et al. 2010; Hayward et al. 2015), our study adds to the growing evidence of sex‐specific parental age effects on long‐term offspring fitness in natural populations. In both wild house sparrows (*Passer domesticus*) and common terns (*Sterna hirundo*) female, but not male, offspring born to older mothers had lower LRS (Bouwhuis et al. 2015; Schroeder et al. 2015). Although differences in life span appear to be driving the lower LRS of female offspring born to older mothers in the Seychelles warbler, in these previous studies differences in annual reproductive success appeared to cause the effect; that is, there was no association between maternal age and offspring life span (Bouwhuis et al. 2015; Schroeder et al. 2015). In contrast to our positive between‐mother age effect observed on the LRS of male offspring, the two previous studies found no maternal age effects on males. However, they found a negative effect of paternal age on male annual reproductive success in the house sparrow and a negative effect of paternal age on male offspring life span in the common tern. Both of these effects translated to reduced LRS of male offspring born to older fathers (Bouwhuis et al. 2015; Schroeder et al. 2015). In contrast, our study found only a positive between‐father age effect on the LRS of male offspring.

The majority of studies in natural populations have found negative effects of increased maternal or paternal age on offspring life span and LRS (Rödel et al. 2009; Bouwhuis et al. 2015; Schroeder et al. 2015), or in one case no effect (Bouwhuis et al. 2010). However, there is also evidence of positive parental age effects. In yellow‐bellied marmots (*Marmota flaviventer*), daughters born to older mothers had greater annual reproductive success, which translated to higher LRS (Kroeger et al. 2020). The authors suggested that younger mothers may lack experience or invest less in offspring, whereas older mothers have more experience or show signs of terminal investment (Kroeger et al. 2020). In a population of semi‐captive Asian elephants (*Elephas maximus*), a complex pattern was observed (Reichert et al. 2019); female offspring survival decreased with increasing maternal age, but daughters from middle‐aged mothers had the lowest LRS. The authors suggest that as middle‐aged mothers have the highest annual reproductive rate and working activity in the timber industry, they may invest less in each offspring (Reichert et al. 2019). In Seychelles warblers, the cross‐sectional positive effect of maternal age on male LRS appeared to be driven by a between‐mother age effect. Although we found no cross‐sectional effect of paternal age, we did find a positive between‐father age effect on male offspring LRS. These positive between‐parent age effects can be driven by any factors driving parents to have an overall higher mean age in the dataset, for example, parents who started to breed at or survived to older ages having male offspring with higher LRS. Importantly, these results show no evidence for within‐parent ageing effects on male offspring life span or LRS.

Associations between parental age and offspring life span could be linked to telomere dynamics. Telomeres, the protective caps at the ends of chromosomes, tend to shorten with age and shorter telomeres are associated with increased mortality risk (Salomons et al. 2009; Wilbourn et al. 2018) including in Seychelles warblers (Barrett et al. 2013). In humans, there is cross‐sectional evidence that sperm telomere length is positively correlated with age and older fathers have offspring with longer telomeres (e.g., Broer et al. 2013). However, evidence from natural vertebrate populations is mixed (Eisenberg 2019). In the Seychelles warbler, there is a weak negative within‐father age at conception effect on offspring telomere length (Sparks et al. 2021). Additionally, there is no evidence for within‐mother age at conception effects on offspring telomere length, which might be expected if there is selection for higher quality oocytes with longer telomeres to be used first (Monaghan et al. 2020). Therefore, the parental age effects on offspring fitness observed in our study are unlikely to be due to parental age at conception effects on offspring telomere length.

Although there is growing evidence of sex‐specific parental age effects on offspring life span and fitness, the mechanisms through which these occur are unknown. Studies have reported different sensitivities between the sexes to poor early‐life environments (Jones et al. 2009). For example, in great tits (*P. major*), natal environmental conditions impacted the life span and breeding success of males but not females (Wilkin and Sheldon 2009). This may be because the sexes respond differently to, or require different levels of, early‐life investment (Jones et al. 2009; Wilkin and Sheldon 2009). Further explanations could include sex‐specific epigenetic inheritance (Broer et al. 2013), age‐ and sex‐specific differences in foraging, offspring provisioning (Weimerskirch 2018), or differential investment in eggs, which could result in complex sex‐specific parental age effects on offspring condition (Bouwhuis et al. 2015).

Despite ample evidence that early‐life environments can affect offspring performance (Monaghan 2008), we found no evidence for environment‐dependent parental age effects on offspring fitness. In the Seychelles warbler, offspring early‐life survival declines with dominant female age when a helper is not present, suggesting that the early‐life social environment is important (Hammers et al. 2021). However, we found no evidence that helper presence, territory quality, or group size impacted the effects of parental age on the fitness of offspring that survived their first year of life (i.e., their most critical period). Only one study has examined the impact of the environment on long‐term parental age effects (Kroeger et al. 2020). In yellow‐bellied marmots, there was a positive association between maternal age and LRS of daughters. However, daughters born to older mothers in favorable environments had greater declines in annual reproductive success with age. This was caused by daughters of older mothers in harsher environments not living long enough to senesce. Further, natal litter size negatively affected daughters born to older but not younger mothers, indicating an offspring number and quality trade‐off for older mothers (Kroeger et al. 2020). Although we did not find evidence for environment‐dependent parental age effects in our study population, the marmot study indicates that parental age effects can be dependent on early‐life environmental conditions.

In the Seychelles warbler, provisioning rates and subsequent juvenile survival decline with dominant female, but not male, age (Hammers et al. 2021). Further, female offspring are more likely to forgo dispersal and help as the dominant female ages (Hammers et al. 2019). This could explain the negative effect of maternal age on the fitness of female, but not male, offspring in our study. However further work is needed to determine the mechanisms by which these sex‐specific parental age effects occur and whether being a helper reduces survival. Although actuarial and reproductive senescence occur in this warbler population (Hammers et al. 2012, 2013; Raj Pant et al. 2020), our study indicates that parental age effects can have longer term effects on offspring fitness. Not investigating such intergenerational effects, including sex‐specific effects, may therefore miss important components of senescence.

A negative effect of increasing parental age on offspring life span may not necessarily have negative consequences for offspring fitness if the reproductive timing of offspring is altered (Monaghan et al. 2020). For instance, in great tits, individual age‐specific reproductive success trajectories were associated with maternal age, but these trajectories equated to similar LRS (Bouwhuis et al. 2010). We found no difference in annual reproductive output in relation to parental age. However, female offspring from older mothers had shorter reproductive life spans and lower LRS than those from younger mothers. In cooperatively breeding species such as the Seychelles warbler, these parental age effects may reveal an additional cost to females of becoming a subordinate if it leads to delayed reproduction. Further, older dominant females are more likely to be displaced from their breeding position by a subordinate than are dominant males (Richardson et al. 2007), which may be adaptive if helping older females to reproduce is less beneficial.

In conclusion, the existence of complex, often sex‐specific, intergenerational effects linked to parental age will have consequences for our understanding of the fitness effects of senescence, and subsequently, evolutionary dynamics. Such effects will also have important ramifications for animal breeding and the conservation of species, particularly where populations are small or age structured and when older individuals are used for breeding or translocations.

## AUTHOR CONTRIBUTIONS

The idea to test the Lansing effect in this population was conceived by AMS, MH, HLD, DSR, TB, and JK. AMS and MH conceived the specific study design, data selection, and methodology. AMS performed all data analyses with input from MH and HLD. HLD, DSR, JK, and TB managed the long‐term Seychelles warbler study system including gaining the relevant funding. HLD constructed the genetic pedigree. AMS wrote the manuscript with input from MH, HLD, and DSR. All authors gave final approval for publication.

## DATA ARCHIVING

Data are available from the Dryad Digital Repository: https://doi.org/10.5061/dryad.7d7wm37zc. All scripts for the analysis are provided at https://github.com/Seychelle‐Warbler‐Project/Sparks_2022_EvolLett.

## Supporting information


**Figure S1**. Histograms of the frequency of offspring lifespan (A‐B) and lifetime reproductive success (measured as the number of assigned offspring in the pedigree who survived to 1 year, C‐D) in the Seychelles warbler for females (A,C) and males (B,D) who survived to one year and had complete lifetime data (A: n=476; B: n=494; C: n=450; D: n=475)
**Figure S2**. Scatterplot of raw data showing the correlation between (genetic) maternal and paternal ages of Seychelles warbler offspring at hatching (n=1321 genetically assigned offspring, 437 mothers and 417 fathers).
**Figure S3**. Within‐individual maternal age effects (‘Delta age mother’) on the lifespan of female offspring in the Seychelles warbler, using within‐subject centering (van de Pol and Wright 2009)
**Figure S4**. Within and between genetic parental age effects on the LRS of female (A) and male (B‐C) offspring in the Seychelles warbler using within‐subject centering (van de Pol and Wright 2009)
**Figure S5**. Scatterplot of raw data showing the correlation between lifespan and lifetime reproductive success (based on genetically assigned offspring) in the Seychelles warbler for all individuals who survived to one year and had complete lifetime data (n=925)
**Figure S6**. Interaction plot describing the marginally significant interaction between the age of the genetic father and territory quality on female offspring lifetime reproductive success (LRS) in the Seychelles warbler (Table S11)
**Figure S7**. Interaction plot describing the marginally significant interaction between the age of the genetic father and territory quality on female offspring lifetime reproductive success (LRS) in the Seychelles warbler (Table S11).
**Figure S8**. Interaction plot describing the marginally significant interaction between the age of the dominant male and territory quality on female offspring lifetime reproductive success (LRS) in the Seychelles warbler (Table S11).
**Figure S9**. Interaction plot describing the marginally significant interaction between the age of the dominant male and territory quality on female offspring lifetime reproductive success (LRS) in the Seychelles warbler (Table S11).
**Table S1**. Generalised linear mixed model (GLMM) results investigating associations between the status of the female and provisioning rate (feed counts) in the Seychelles warbler
**Table S2**. Generalised linear mixed model (GLMM) results investigating the associations between parental age and offspring lifespan and LRS where age of the father and age of the dominant male were included in separate models
**Table S3**. Cox proportional hazards mixed effects model results for parental age effects on offspring lifespan, for males and females separately, in the Seychelles warbler
**Table S4**. Generalised linear mixed model results investigating between versus within maternal and paternal age effects on offspring lifespan, in each offspring sex separately, in the Seychelles warbler using the within‐subject centering method (van de Pol and Wright 2009).
**Table S5**. Generalised linear mixed model results investigating between versus within maternal and paternal age effects on offspring lifespan, in each sex separately, in the Seychelles warbler, using the within‐subject centering method (van de Pol and Wright 2009).
**Table S6**. Significance of maternal and paternal age interactions, quadratic parental age effects and environment by parental age interactions on female offspring and male offspring lifespan, in the Seychelles warbler using a GLMM
**Table S7**. Significance of maternal and paternal age interactions, quadratic parental age effects and environment by parental age interactions on female offspring and male offspring lifespan, in the Seychelles warbler, in the Cox proportional hazards mixed effects model
**Table S8**. Generalised linear mixed model results investigating associations between parental age and offspring reproductive lifespan in the Seychelles warbler for each sex separately
**Table S9**. Generalised linear mixed model results investigating between versus within maternal and paternal age effects on offspring LRS in each offspring sex separately in the Seychelles warbler using the within‐subject centering method (van de Pol and Wright 2009)
**Table S10**. Generalised linear mixed model results investigating between‐ versus within‐ maternal and paternal age effects on offspring lifetime reproductive success, in each sex separately, in the Seychelles warbler using the within‐subject centering method (van de Pol and Wright 2009)
**Table S11**. Significance of maternal and paternal age interactions, quadratic parental age effects and environment by parental age interactions on female offspring and male offspring lifetime reproductive success in the Seychelles warbler
**Table S12**. Generalised linear mixed model results investigating parental age effects on offspring annual reproductive success (measured as the number of offspring in the pedigree who survived to 1 year), in each sex separately, in the Seychelles warbler
**Table S13**. Generalised linear mixed model results investigating between versus within maternal and paternal age effects on female offspring lifespan in the Seychelles warbler, using the within‐subject centering method (van de Pol and Wright 2009)
**Table S14**. Generalised mixed model results investigating between versus within maternal and paternal age effects on female offspring lifetime reproductive success in the Seychelles warbler using the within‐subject centering method (van de Pol and Wright 2009)
