# Supplementary material for: Sex‐dependent effects of parental age on offspring fitness in a cooperatively breeding bird
Source: Evol Lett. 2022 Nov 16;6(6):438–49. doi: 10.1002/evl3.300 (PMC9783413; doi:10.1002/evl3.300)
Supplement: Supplementary file 1 — Figure S1. Histograms of the frequency of offspring lifespan (A‐B) and lifetime reproductive success (measured as the number of assigned offspring in the pedigree who survived to 1 year, C‐D) in the Seychelles warbler for females (A,C) and males (B,D) who survived to one year and had complete lifetime data (A: n=476; B: n=494; C: n=450; D: n=475) Figure S2. Scatterplot of raw data showing the correlation between (genetic) maternal and paternal ages of Seychelles warbler offspring at hatching (n=1321 genetically assigned offspring, 437 mothers and 417 fathers). Figure S3. Within‐individual maternal age effects (‘Delta age mother’) on the lifespan of female offspring in the Seychelles warbler, using within‐subject centering (van de Pol and Wright 2009) Figure S4. Within and between genetic parental age effects on the LRS of female (A) and male (B‐C) offspring in the Seychelles warbler using within‐subject centering (van de Pol and Wright 2009) Figure S5. Scatterplot of raw data showing the correlation between lifespan and lifetime reproductive success (based on genetically assigned offspring) in the Seychelles warbler for all individuals who survived to one year and had complete lifetime data (n=925) Figure S6. Interaction plot describing the marginally significant interaction between the age of the genetic father and territory quality on female offspring lifetime reproductive success (LRS) in the Seychelles warbler (Table S11) Figure S7. Interaction plot describing the marginally significant interaction between the age of the genetic father and territory quality on female offspring lifetime reproductive success (LRS) in the Seychelles warbler (Table S11). Figure S8. Interaction plot describing the marginally significant interaction between the age of the dominant male and territory quality on female offspring lifetime reproductive success (LRS) in the Seychelles warbler (Table S11). Figure S9. Interaction plot describing the marginally significant interaction between [file EVL3-6-438-s001.pdf]

Supplementary material for:

Sex-dependent effects of parental age on offspring fitness in a cooperatively breeding bird

Alexandra M. Sparks, Martijn Hammers, Jan Komdeur, Terry Burke,

David S. Richardson, Hannah L. Dugdale

Supplementary methods

Territory quality

Territory quality was calculated using $A*\sum(Cx*Ix)$, where A is the territory size (in hectares), Cx is the amount of foliage cover for tree species x, and Ix is the mean monthly insect density for tree species x per unit leaf area in dm^2 (following Komdeur 1992; van de Crommenacker et al. 2011). Where territory quality was not available for that breeding season (n=384 of 1366 unique breeding season/territory combinations), we used the mean territory quality of the previous and next field period of the same season (south-east or north-west monsoon) to account for temporal variation in territory quality (Brouwer et al. 2006, 2012; Raj Pant et al. 2019). This method is a good proxy for territory quality given there is little variation in the number of territories over time and territories are spatially stable over time (Brouwer et al. 2006, 2012). This allowed us to maximise the sample size to estimate parental age effects while accounting for other early life environmental effects in the model.

Reproductive lifespan

We tested whether any parental age effects on lifespan translated to differences in reproductive lifespan. We calculated reproductive lifespan as the difference between age at last and first breeding defined by the first and last year an individual had offspring in the genetic pedigree. We included all individuals who survived to one year of age, were not translocated, and whose last seen year was before 2018 (last year of the pedigree). Analyses were performed using a zero-inflated poisson GLMM in glmmTMB (Brooks et al. 2017) with the same model structure as for the lifespan GLMM. However, we did not test quadratic

parental age effects or interactions between parental age and early life environmental variables in these models as there was no evidence for these in the lifespan models (see Results).

Annual reproductive success

In addition to lifespan, parental age effects on offspring LRS may be caused by differences in the annual reproductive output of offspring. Annual reproductive success of offspring was calculated as the number of offspring produced in a year who survived to one year of age. This was calculated for all individuals who survived to one year of age and had complete LRS data. Analyses were performed using a poisson GLMM in glmmTMB. We included age (linear and squared), lifespan, ages of the mother, father, and dominant male, hatch year, territory quality, group size and the presence of helpers (factor) or siblings (factor) as fixed effects and individual identity and year as random effects. Since only linear parental age effects were found in the main models of LRS and there was no evidence for environment-dependent parental age effects (see Results), we did not test quadratic parental age effects or interactions between parental age and early life environmental variables in these models.

Within-subject centering

To investigate whether parental age effects were driven by within- or between-individual parental age effects we used within-subject centering (van de Pol and Wright 2009). We included mean age per parent (between-individual parental age effects, e.g. testing for selective disappearance of lower-quality individuals) and the deviation from the mean age of

the parent (testing for within-individual parental age effects) in the GLMMs of LRS and lifespan. To test whether the within- and between-individual parental age slopes were significantly different from each other, we included age of the parent (within-individual parental age effects) and mean age of the parent (difference between the within- and between-individual parental age slopes) as predictors in a second model. Using this parameterisation of the model, the significance of mean age indicates that the within and between-individual parental age slopes in the first model are significantly different from each other (van de Pol and Wright 2009). Since only linear and not quadratic parental age effects were found in the main models of LRS and lifespan (see Results), we only separated out linear parental age effects using within-subject centering. These models also included the fixed effects of hatch year, territory quality, group size, helper presence (factor) and sibling presence (factor). Maternal, paternal and dominant male identity and hatch year were included as random effects. Interactions between parental age and early life environmental variables were not tested in these models as these were not significant in the main models of LRS and lifespan (see Results).

Supplementary results

When parental age effects on offspring lifespan were separated into within- versus between-individual parental age effects there was a significant negative within-individual maternal age effect on female offspring lifespan (Table S4, Figure S3). The within-individual maternal age slope was significantly more negative than the between-individual maternal age slope (Table S4-5). There were no within- or between-individual maternal age effects on male offspring

lifespan, and no within- or between-individual paternal age effects on offspring lifespan of either sex (Table S4).

Parental age effects on offspring LRS were separated into within- versus between-individual parental age effects. There was a significant negative within-individual maternal age effect on female offspring LRS (Table S9, Figure S4A). As individual mothers aged, the female offspring they produced had lower LRS (Table S9, Figure S4A). The within- versus between-individual maternal age slopes were not significantly different from each other (Table S10). There was also a significant positive between-individual maternal age effect and a marginally significant positive between-individual paternal age effect on male offspring LRS (Table S9, Figure S4B-C). This shows that, cross-sectionally, mothers and fathers that survived to, or started to breed at, older ages had male offspring with higher LRS (Table S9, Figure S4B-C). However, there was no difference in the within versus between slopes for either maternal or paternal age for male offspring (Table S10).

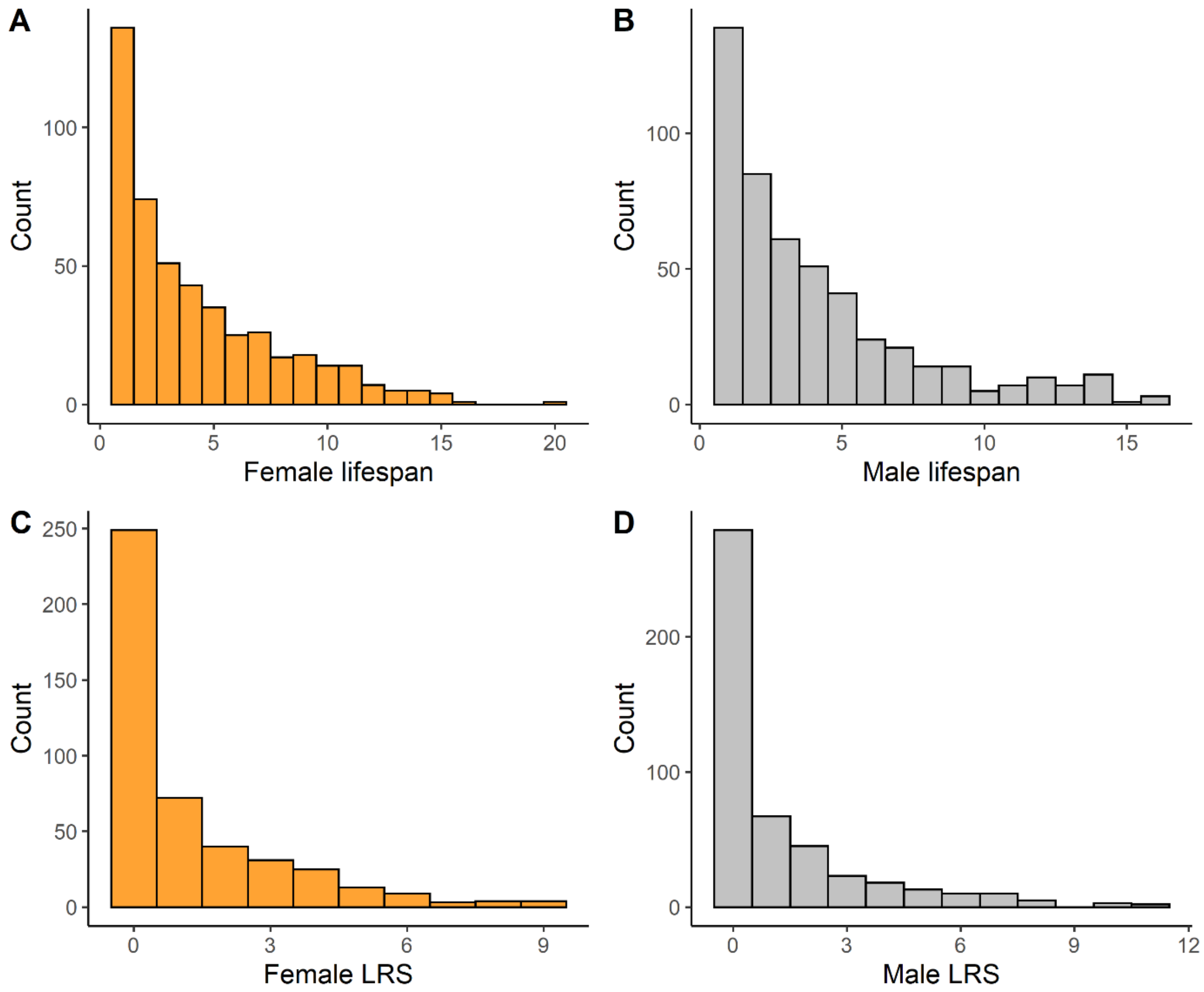

Figure S1. Histograms of the frequency of offspring lifespan (A-B) and lifetime reproductive success (measured as the number of assigned offspring in the pedigree who survived to 1 year, C-D) in the Seychelles warbler for females (A,C) and males (B,D) who survived to one year and had complete lifetime data (A: n=476; B: n=494; C: n=450; D: n=475).

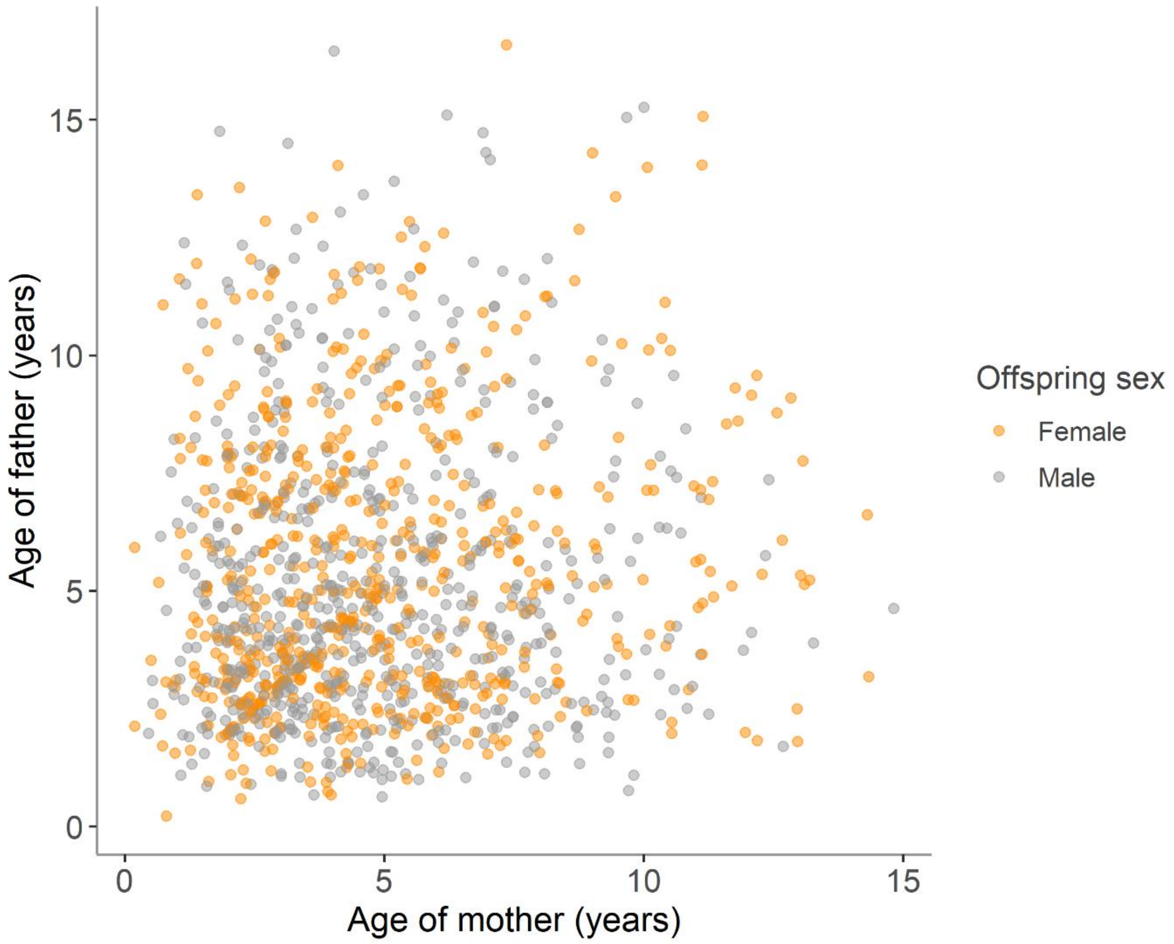

Figure S2. Scatterplot of raw data showing the correlation between (genetic) maternal and paternal ages of Seychelles warbler offspring at hatching (n=1321 genetically assigned offspring, 437 mothers and 417 fathers).

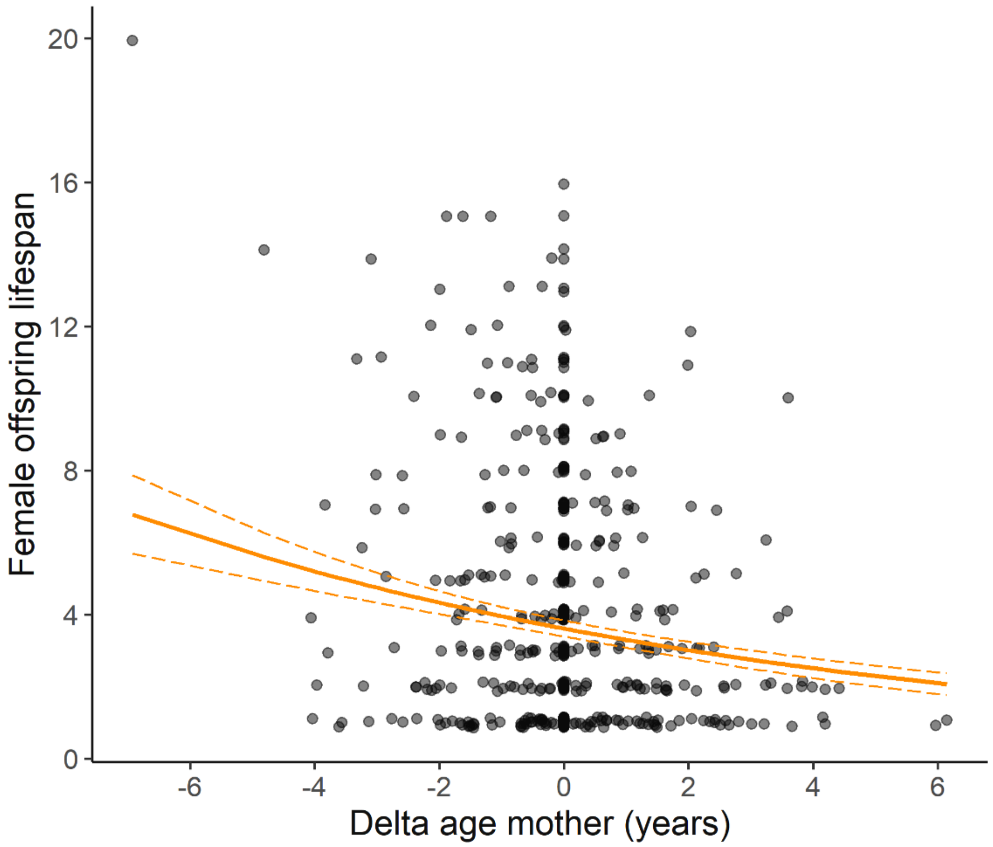

Figure S3. Within-individual maternal age effects ('Delta age mother') on the lifespan of female offspring in the Seychelles warbler, using within-subject centering (van de Pol and Wright 2009). The solid line is the GLMM prediction with mean values for all other continuous fixed effects in the model and the dashed lines indicate standard errors (Table S4). Dots show the raw data points. Lifespan values are integers but are jittered to show overlapping values. Model estimates (Table S4) are similar without the outlier (the offspring with a lifespan of 20 years) (Table S13).

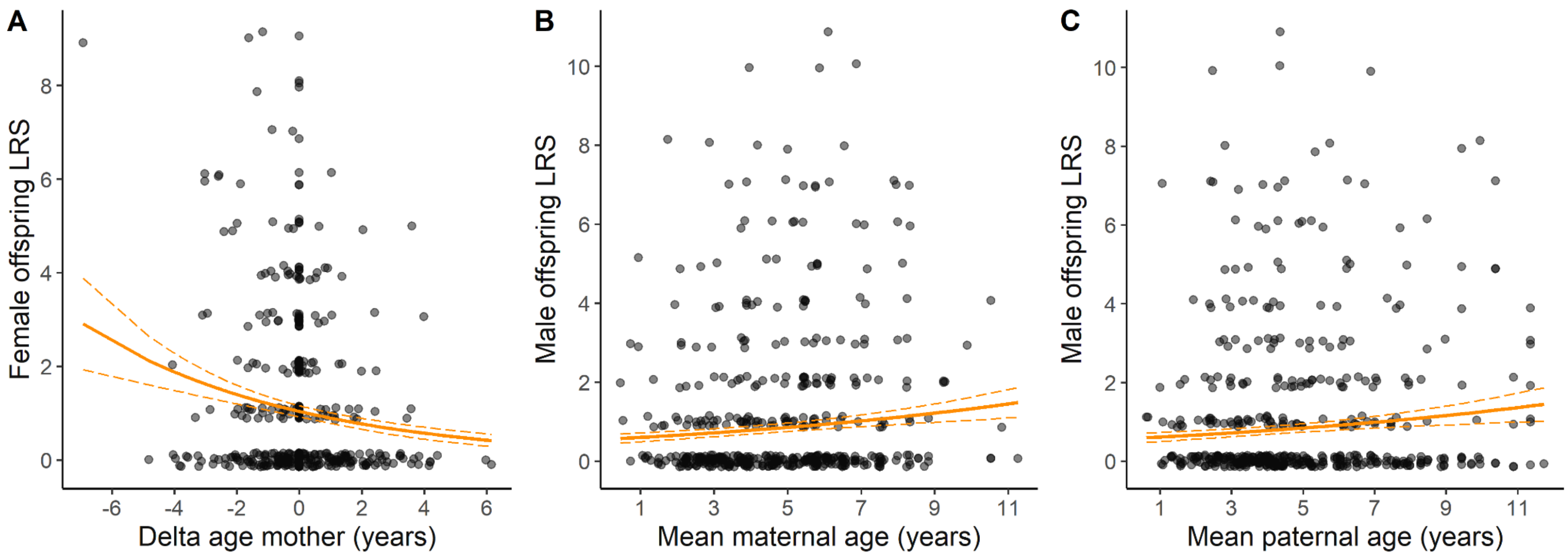

Figure S4. Within and between genetic parental age effects on the LRS of female (A) and male (B-C) offspring in the Seychelles warbler using within-subject centering (van de Pol and Wright 2009). Lines indicate GLMM predictions with mean values for all other continuous fixed effects in the model and dashed lines indicate standard errors (Table S9). Dots show the raw data points. LRS values are integers but are jittered to show overlapping values. Model estimates (Table S9) are similar without the outlier in Figure S4A (Table S14).

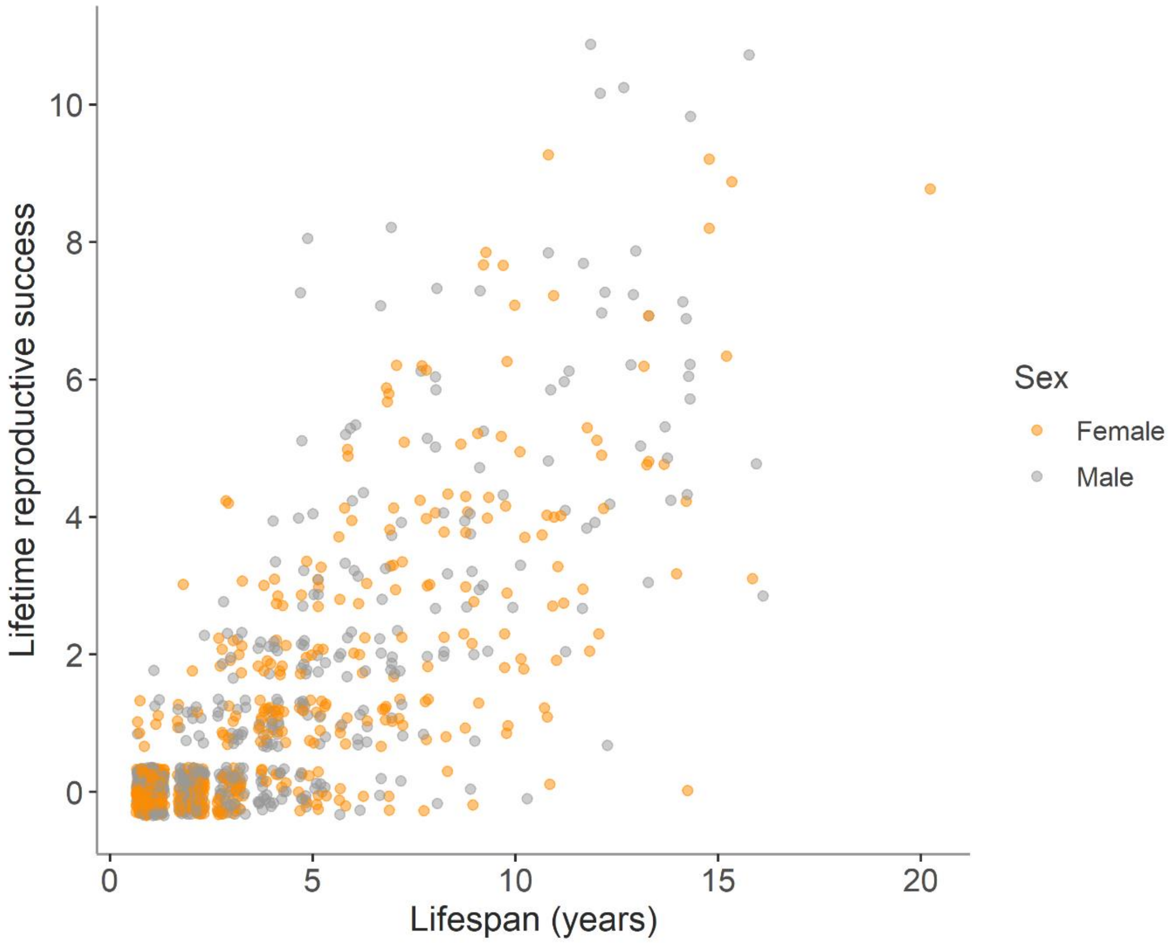

Figure S5. Scatterplot of raw data showing the correlation between lifespan and lifetime reproductive success (based on genetically assigned offspring) in the Seychelles warbler for all individuals who survived to one year and had complete lifetime data (n=925). Lifespan and LRS are integers but jittered to minimise overplotting.

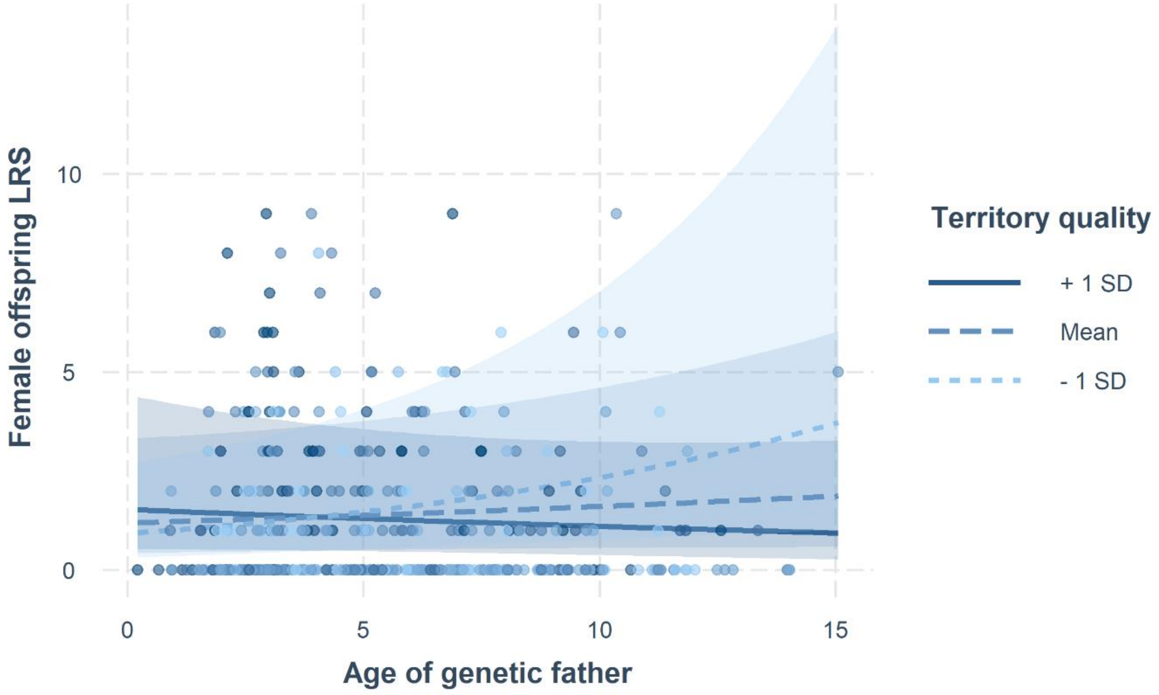

Figure S6. Interaction plot describing the marginally significant interaction between the age of the genetic father and territory quality on female offspring lifetime reproductive success (LRS) in the Seychelles warbler (Table S11). Dots show the raw data points, colour coded by territory quality (lighter = lower territory quality, darker = higher territory quality). Lines show model predictions and shaded areas indicate 95% confidence intervals for the relationship of the age of the genetic father and female offspring LRS on three different levels of territory quality (mean territory quality +/- 1 standard deviation from the mean, see legend). The interaction is based on limited data points (N=38) for older males (>10 years) so there are large error estimates (95% confidence intervals) around these values indicated by the shaded areas. The interaction plot between the age of the dominant male and territory quality on female offspring LRS showed a similar pattern (Figure S8).

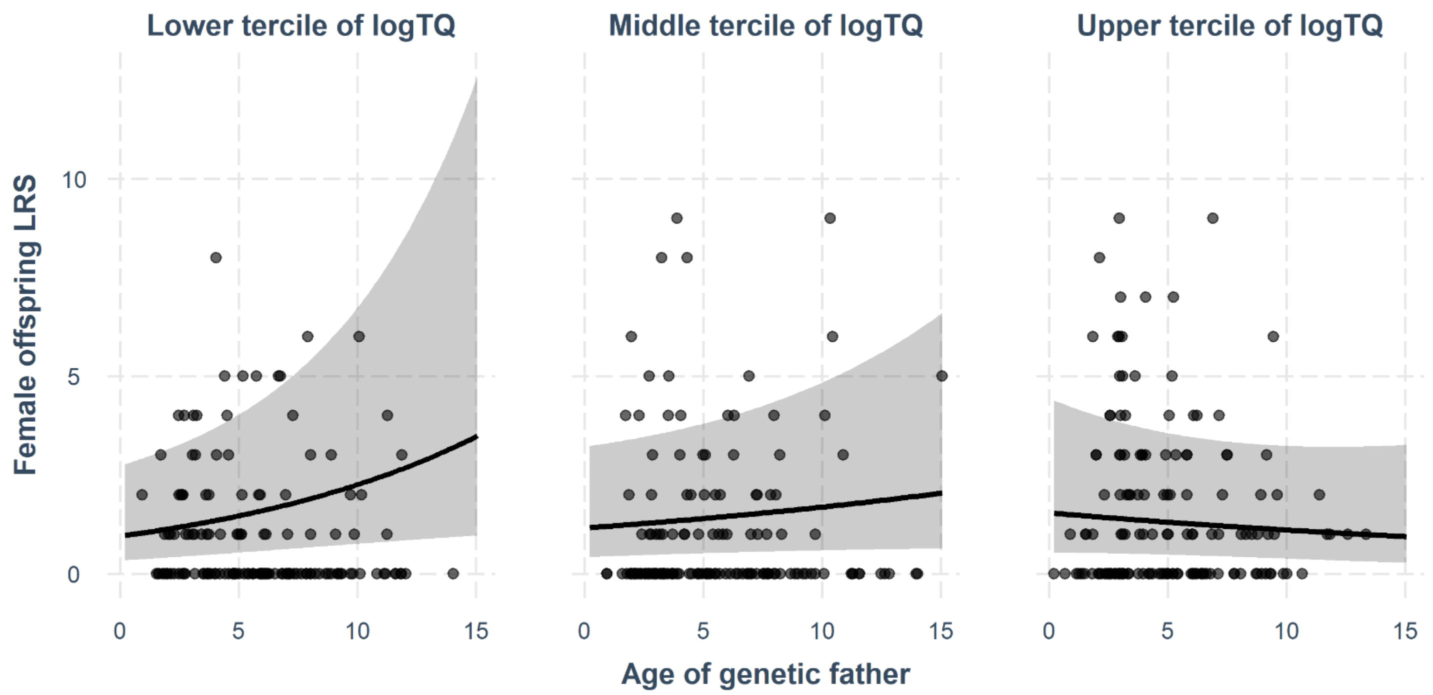

Figure S7. Interaction plot describing the marginally significant interaction between the age of the genetic father and territory quality on female offspring lifetime reproductive success (LRS) in the Seychelles warbler (Table S11). This multi-panel plot is split into three approximately equal-sized groups with the lowest third, middle third and highest third of the territory quality data accordingly. Dots show the raw data points. The lines show the estimated relationship between the age of the genetic father and female offspring LRS within each tercile of the territory quality data with the shaded area indicating the 95% confidence interval. The slopes of each line are based on a few points for older (>10 years) males (N=38) such that there are large error estimates (95% confidence intervals) around these values indicated by the shaded areas. The interaction plot between the age of the dominant male and territory quality on female offspring LRS showed a similar pattern (Figure S9).

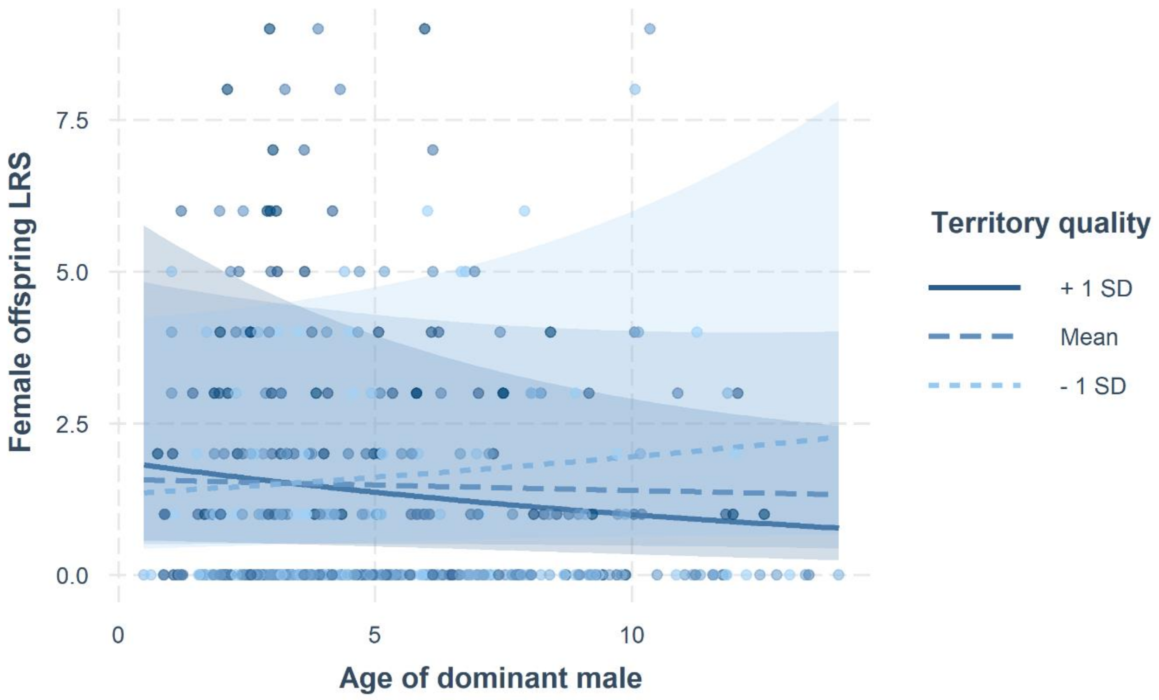

Figure S8. Interaction plot describing the marginally significant interaction between the age of the dominant male and territory quality on female offspring lifetime reproductive success (LRS) in the Seychelles warbler (Table S11). Dots show the raw data points, colour coded by territory quality (lighter = lower territory quality, darker = higher territory quality). Lines show model predictions and shaded areas indicate 95% confidence intervals for the relationship of the age of the dominant male and female offspring LRS on three different levels of territory quality (mean territory quality +/- 1 standard deviation from the mean, see legend). The interaction is based on limited data points (N=34) for older males (>10 years) so there are large error estimates (95% confidence intervals) around these values indicated by the shaded areas.

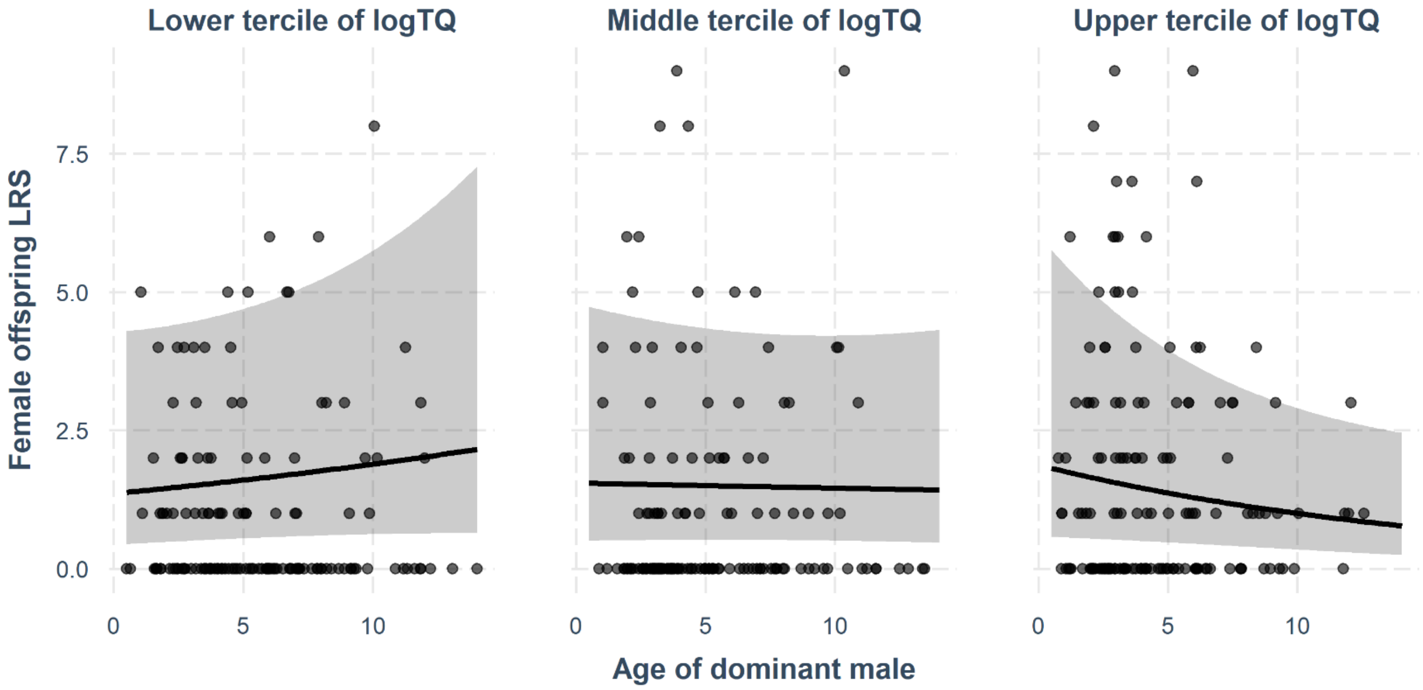

Figure S9. Interaction plot describing the marginally significant interaction between the age of the dominant male and territory quality on female offspring lifetime reproductive success (LRS) in the Seychelles warbler (Table S11). This multi-panel plot is split into three approximately equal-sized groups with the lowest third, middle third and highest third of the territory quality data accordingly. Dots show the raw data points. The lines show the estimated relationship between the age of the dominant male and female offspring LRS within each tercile of the territory quality data with the shaded area indicating the 95% confidence interval. The slopes of each line are based on a few points for older (>10 years) males (N=34) such that there are large error estimates (95% confidence intervals) around these values indicated by the shaded areas.

Table S1. Generalised linear mixed model (GLMM) results investigating associations between the status of the female and provisioning rate (feed counts), in the Seychelles warbler. The model included nest watch data from nests where at least one of the chicks (brood sizes 1-3) in the nest was from a subordinate female (using the pedigree) and included nest watches where there were ≥90% of birds identified. The response variable was the total number of feed counts of the subordinate and dominant female for each nest watch. To account for differences in length of nest watches (usually 60 minutes), an offset of the nest watch duration (logged) was added. The zero-inflated Poisson GLMM was run in glmmTMB v1.0.2.1 (Brooks et al. 2017). Included are the parameter estimates (estimate), their standard errors (SE), and the significance of fixed effects based on a likelihood ratio test (LRT, P) where d.f.=1. Reference levels for the female status was dominant and for the watch type was feeding. There was no significant difference in provisioning rates between the cobreeding subordinate female and dominant female. N=49 nest watch IDs, each with a provisioning count for the dominant female and subordinate female (N=98 rows). Significant fixed effects are highlighted in bold.

	**Provisioning rate**			
variables	estimate	SE	LRT	P
fixed effects				
intercept	-2.167	0.181		
status of female (subordinate female)	0.076	0.094	0.627	0.428
brood size	0.133	0.107	1.537	0.215
watch type (feeding and incubating)	**-0.431**	**0.120**	**12.124**	**<0.001**
random effects				
NestWatchID/NestID	0.052			
NestID	<0.001			
BirdID	0.034			
Observer	<0.001			
Year	0.073			

Table S2. Generalised linear mixed model (GLMM) results investigating the associations between parental age and offspring lifespan and LRS where age of the father and age of the dominant male were included in separate models. Included are the estimated effects (estimate), standard errors (SEs), and significance of fixed effects based on a likelihood ratio test (LRT; P-value) where df=1. Model 1 indicates where age of the mother and father are included and not dominant male age or dominant male ID as a random effect, and model 2 indicates where age of the mother and dominant male are included and not age of the father or father ID as a random effect. Model structure otherwise is the same as that in Table 1 and 2. Although estimates may differ, overall conclusions of the models based on significance are the same as to when age of the father and age of the dominant male are included in the same model.

	**Lifespan – female offspring**				
	Fixed effect	estimate	SE	LRT	P
Model 1	Age of mother	-0.238	0.075	10.325	0.001
	Age of father	0.034	0.076	0.200	0.655
Model 2	Age of mother	-0.280	0.074	14.342	<0.001
	Age of dominant male	0.078	0.073	1.146	0.284
	**Lifespan – male offspring**				
	Fixed effect	estimate	SE	LRT	P
Model 1	Age of mother	0.101	0.066	2.334	0.127
	Age of father	0.013	0.074	0.032	0.858
Model 2	Age of mother	0.088	0.066	1.761	0.185
	Age of dominant male	0.110	0.070	2.431	0.119
	**LRS – female offspring**				
	Fixed effect	estimate	SE	LRT	P
Model 1	Age of mother	-0.427	0.153	7.894	0.005
	Age of father	0.108	0.143	0.573	0.449
Model 2	Age of mother	-0.420	0.148	7.947	0.005
	Age of dominant male	-0.039	0.139	0.082	0.775
	**LRS – male offspring**				
	Fixed effect	estimate	SE	LRT	P
Model 1	Age of mother	0.326	0.139	5.555	0.018
	Age of father	0.277	0.158	0.573	0.449
Model 2	Age of mother	0.301	0.141	4.582	0.032
	Age of dominant male	0.226	0.151	0.082	0.775

Table S3. Cox proportional hazards mixed effects model results for parental age effects on offspring lifespan, for males and females separately, in the Seychelles warbler. Included are the parameter estimates (estimate) and their standard errors (SE), the exponentiated coefficient (exp(est)) which represents multiplication effects on the hazard and the significance of fixed effects based on a likelihood ratio test (LRT, P) where d.f.=1. Significance of dropped squared parental age variables, and the environment by parental age interactions are shown in Table S7. Significant fixed effects are in bold.

	**Female offspring lifespan**					**Male offspring lifespan**				
	n=627					n=670				
variables	estimate	SE	exp(est)	LRT	P	estimate	SE	exp(est)	LRT	P
fixed effects										
age of mother	**0.034**	**0.017**	**1.035**	**3.871**	**0.049**	0.010	0.018	1.010	0.2791	0.597
age of father	0.001	0.018	1.001	0.013	0.909	0.003	0.020	1.003	0.023	0.880
age of dominant male	0.002	0.019	1.002	0.018	0.895	-0.037	0.020	0.963	3.6529	0.056
territory quality	**-0.265**	**0.122**	**0.767**	**4.707**	**0.030**	-0.136	0.144	0.872	0.8909	0.345
group size	0.109	0.069	1.115	2.360	0.125	0.140	0.072	1.150	3.651	0.056
helper presence (y)	0.222	0.130	1.248	2.866	0.090	-0.242	0.139	0.785	3.1135	0.078
sibling presence (y)	-0.011	0.151	0.989	0.006	0.941	-0.161	0.143	0.851	1.2858	0.257
random effects										
maternal ID	<0.001					<0.001				
paternal ID	<0.001					<0.001				
dominant male ID	<0.001					<0.001				
hatch year	0.020					0.096				

Table S4. Generalised linear mixed model results investigating between versus within maternal and paternal age effects on offspring lifespan, in each offspring sex separately, in the Seychelles warbler using the within-subject centering method (van de Pol and Wright 2009). Included are the estimated effects (estimate), standard errors (SEs), and significance of fixed effects based on a likelihood ratio test (LRT; P-value) where df=1. The model investigates within-parental age effects (deviation from the mean age of the parent) and between-parental age effects (mean age for each parent). Significant fixed effects are in bold.

	**Female offspring lifespan**				**Male offspring lifespan**			
	n=467				n=485			
variables	estimate	SE	LRT	P	estimate	SE	LRT	P
fixed effects								
intercept	1.284	0.062			1.203	0.070		
between-maternal age	-0.097	0.090	1.140	0.286	0.127	0.095	1.793	0.181
within-maternal age	**-0.508**	**0.121**	**17.503**	**<0.001**	0.092	0.096	0.920	0.338
between-paternal age	0.004	0.110	0.001	0.973	0.045	0.104	0.189	0.664
within-paternal age	-0.157	0.134	1.361	0.243	-0.198	0.128	2.437	0.119
between-dominant male age	0.113	0.094	1.437	0.231	0.158	0.099	2.570	0.109
within-dominant male age	0.252	0.146	2.974	0.085	0.102	0.131	0.604	0.437
hatch year	**-0.525**	**0.112**	**19.000**	**<0.001**	**-0.514**	**0.138**	**12.252**	**<0.001**
territory quality	0.022	0.080	0.075	0.784	0.150	0.093	2.645	0.104
group size	-0.104	0.077	1.825	0.177	-0.065	0.081	0.654	0.419
helper presence (y)	-0.153	0.092	2.806	0.094	-0.140	0.101	1.936	0.164
sibling presence (y)	-0.069	0.103	0.446	0.504	0.142	0.100	1.991	0.158
random effects								
maternal ID	0.089				0.088			
paternal ID	0.170				0.081			
dominant male ID	0.004				0.069			
hatch year	0.022				0.054			

Table S5. Generalised linear mixed model results investigating between- versus within- maternal and paternal age effects (in years) on offspring lifespan, in each sex separately, in the Seychelles warbler, using the within-subject centering method (van de Pol and Wright 2009). Included are the estimated effects (estimate), standard errors (SEs), and significance of fixed effects based on a likelihood ratio test (LRT; P-value) where df=1. The model investigates whether the within- and between-parental age slopes from Table S4 are significantly different from each other (mean age represents the difference between the slopes and is significant if the slopes are significantly different from each other). The age component is now equivalent to the within-parental age slope from Table S4. Significant fixed effects are highlighted in bold.

	**Female offspring lifespan**				**Male offspring lifespan**			
	n=467				n=485			
variables	estimate	SE	LRT	P	estimate	SE	LRT	P
fixed effects								
intercept	1.284	0.062			1.204	0.070		
mean maternal age	**0.412**	**0.146**	**7.939**	**0.005**	0.035	0.134	0.069	0.794
maternal age	**-0.508**	**0.121**	**17.503**	**<0.001**	0.092	0.096	0.920	0.338
mean paternal age	0.161	0.166	0.928	0.335	0.244	0.154	2.503	0.114
paternal age	-0.157	0.134	1.361	0.243	-0.198	0.128	2.437	0.119
mean dominant male age	-0.139	0.169	0.675	0.411	0.056	0.158	0.126	0.723
dominant male age	0.252	0.146	2.974	0.085	0.102	0.131	0.604	0.437
hatch year	**-0.525**	**0.112**	**19.000**	**<0.001**	**-0.514**	**0.138**	**12.252**	**<0.001**
territory quality	0.022	0.080	0.075	0.784	0.150	0.093	2.645	0.104
group size	-0.104	0.077	1.825	0.177	-0.065	0.081	0.654	0.419
helper presence (y)	-0.153	0.092	2.806	0.094	-0.140	0.101	1.936	0.164
sibling presence (y)	-0.069	0.103	0.446	0.504	0.142	0.100	1.991	0.158
random effects								
maternal ID	0.089				0.088			
paternal ID	0.170				0.081			
dominant male ID	0.004				0.069			
hatch year	0.022				0.054			

Table S6. Significance of maternal and paternal age interactions, quadratic parental age effects and environment by parental age interactions on female offspring and male offspring lifespan, in the Seychelles warbler using a GLMM. Significance was determined by a likelihood ratio test (LRT, P) where df=1, by adding the variable to the minimal model (Table 1).

	**Female offspring lifespan**			**Male offspring lifespan**		
variables	LRT	df	P	LRT	df	P
age of mother^2	0.001	1	0.970	2.523	1	0.112
age of father^2	0.483	1	0.487	0.808	1	0.369
age of dominant male^2	1.547	1	0.214	1.347	1	0.246
territory quality*maternal age	0.407	1	0.524	0.290	1	0.590
territory quality*paternal age	0.052	1	0.820	0.295	1	0.587
territory quality*dominant male age	0.121	1	0.728	0.095	1	0.758
group size*maternal age	2.650	1	0.104	1.164	1	0.281
group size*paternal age	0.126	1	0.722	2.082	1	0.149
group size*dominant male age	1.407	1	0.236	1.563	1	0.211
helper*maternal age	0.002	1	0.968	0.057	1	0.812
helper*paternal age	2.163	1	0.141	0.838	1	0.360
helper*dominant male age	0.326	1	0.568	1.122	1	0.290
age of mother*age of father	0.131	1	0.717	1.144	1	0.285
age of mother*age of dominant male	2.738	1	0.098	0.100	1	0.753

Table S7. Significance of maternal and paternal age interactions, quadratic parental age effects and environment by parental age interactions on female offspring and male offspring lifespan, in the Seychelles warbler, in the Cox proportional hazards mixed effects model. Significance was determined by a likelihood ratio test (LRT, P) where df=1, by adding the variable to the minimal model (Table S3).

	**Female offspring lifespan**		**Male offspring lifespan**	
variables	LRT	P	LRT	P
age of mother^2	0.008	0.929	0.105	0.746
age of father^2	1.954	0.162	0.152	0.697
age of dominant male^2	0.016	0.899	0.878	0.349
territory quality*maternal age	0.044	0.833	0.420	0.517
territory quality*paternal age	0.124	0.724	1.842	0.175
territory quality*dominant male age	0.063	0.802	0.147	0.702
group size*maternal age	0.748	0.387	0.032	0.859
group size*paternal age	1.311	0.252	0.011	0.916
group size*dominant male age	0.010	0.919	0.381	0.537
helper*maternal age	0.007	0.934	0.784	0.376
helper*paternal age	2.042	0.153	0.692	0.406
helper*dominant male age	0.076	0.783	0.020	0.889
age of mother*age of father	0.425	0.515	0.131	0.717
age of mother*age of dominant male	2.207	0.137	0.539	0.463

Table S8. Generalised linear mixed model results investigating associations between parental age (in years) on offspring reproductive lifespan in the Seychelles warbler for each sex separately. Included are the parameter estimates (estimate), their standard errors (SE), and the significance of fixed effects based on a likelihood ratio test (LRT, P) where d.f.=1. n refers to the number of offspring with full LRS data. Significant fixed effects are highlighted in bold.

	**Female offspring reproductive lifespan**				**Male offspring reproductive lifespan**			
	n=441				n=466			
variables	estimate	SE	LRT	P	estimate	SE	LRT	P
fixed effects								
intercept	0.722	0.125			0.338	0.151		
age of mother	**-0.467**	**0.147**	**9.932**	**0.002**	0.262	0.139	3.571	0.059
age of father	0.118	0.155	0.575	0.448	0.102	0.187	0.295	0.587
age of dominant male	-0.049	0.156	0.098	0.755	0.128	0.168	0.586	0.444
hatch year	-1.353	0.233	32.013	<0.001	-1.481	0.294	21.230	<0.001
territory quality	-0.182	0.138	1.721	0.190	0.230	0.213	1.193	0.275
group size	**-0.277**	**0.140**	**3.978**	**0.046**	0.068	0.161	0.176	0.675
helper presence (y)	-0.372	0.195	3.590	0.058	-0.202	0.203	1.002	0.317
sibling presence (y)	-0.182	0.181	1.002	0.317	-0.183	0.213	0.711	0.399
random effects								
maternal ID	0.135				0.095			
paternal ID	0.109				0.382			
dominant male ID	0.147				0.117			
hatch year	0.067				0.173			

Table S9. Generalised linear mixed model results investigating between versus within maternal and paternal age effects on offspring LRS in each offspring sex separately in the Seychelles warbler, using the within-subject centering method (van de Pol and Wright 2009). Included are the estimated effects (estimate), standard errors (SEs), and significance of fixed effects based on a likelihood ratio test (LRT; P-value) where df=1. The model investigates within-parental age effects (deviation from the mean age of the parent) and between-parental age effects (mean age for each parent). Significant fixed effects are in bold.

	**Female offspring LRS** n=441				**Male offspring LRS** n=466			
variables	estimate	SE	LRT	P	estimate	SE	LRT	P
fixed effects								
intercept	0.513	0.131			0.345	0.143		
between-maternal age	-0.298	0.166	3.197	0.074	**0.442**	**0.176**	**6.424**	**0.011**
within-maternal age	**-0.835**	**0.261**	**9.640**	**0.002**	0.139	0.219	0.402	0.526
between-paternal age	0.046	0.178	0.067	0.796	**0.461**	**0.220**	**4.383**	**0.036**
within-paternal age	0.291	0.278	1.103	0.294	-0.071	0.277	0.067	0.796
between-dominant male age	-0.140	0.170	0.690	0.406	-0.134	0.192	0.491	0.484
within-dominant male age	0.025	0.301	0.007	0.935	0.324	0.307	1.129	0.288
hatch year	**-1.076**	**0.227**	**23.569**	**<0.001**	**-1.119**	**0.272**	**16.840**	**<0.001**
territory quality	-0.136	0.142	0.927	0.336	0.219	0.199	1.211	0.271
group size	0.004	0.151	0.001	0.981	-0.097	0.175	0.304	0.581
helper presence (y)	**-0.611**	**0.201**	**9.336**	**0.002**	-0.279	0.222	1.643	0.200
sibling presence (y)	-0.109	0.189	0.333	0.564	0.070	0.204	0.118	0.732
random effects								
maternal ID	0.100				0.153			
paternal ID	0.090				0.234			
dominant male ID	0.046				<0.001			
hatch year	0.064				0.085			

Table S10. Generalised linear mixed model results investigating between- versus within- maternal and paternal age effects (in years) on offspring lifetime reproductive success, in each sex separately, in the Seychelles warbler using the within-subject centering method (van de Pol and Wright 2009). Included are the estimated effects (estimate), standard errors (SEs), and significance of fixed effects based on a likelihood ratio test (LRT; P-value) where df=1. The model investigates whether the within and between parental age slopes from Table S9 are significantly different from each other (mean age represents the difference between the slopes and is significant if the slopes are significantly different from each other). The age component is now equivalent to the within parental age slope from Table S9. Significant fixed effects are highlighted in bold.

	**Female offspring LRS** n=441 females				**Male offspring LRS** n=466 males			
variables	estimate	SE	LRT	P	estimate	SE	LRT	P
fixed effects								
intercept	0.513	0.131			0.345	0.143		
mean maternal age	0.537	0.289	3.344	0.067	0.303	0.274	1.219	0.270
maternal age	**-0.835**	**0.261**	**9.640**	**0.002**	0.139	0.219	0.402	0.526
mean paternal age	-0.245	0.303	0.659	0.417	0.533	0.345	2.449	0.118
paternal age	0.291	0.278	1.103	0.294	-0.071	0.277	0.067	0.796
mean dominant male age	-0.164	0.322	0.260	0.610	-0.459	0.379	1.493	0.222
dominant male age	0.025	0.301	0.007	0.935	0.324	0.307	1.129	0.288
hatch year	**-1.076**	**0.227**	**23.569**	**<0.001**	**-1.119**	**0.272**	**16.840**	**<0.001**
territory quality	-0.136	0.142	0.927	0.336	0.219	0.199	1.211	0.271
group size	0.004	0.151	0.001	0.981	-0.097	0.175	0.304	0.581
helper presence (y)	**-0.611**	**0.201**	**9.336**	**0.002**	-0.279	0.222	1.643	0.200
sibling presence (y)	-0.109	0.189	0.333	0.564	0.070	0.204	0.118	0.732
random effects								
maternal ID	0.100				0.153			
paternal ID	0.090				0.234			
dominant male ID	0.046				<0.001			
hatch year	0.064				0.085			

Table S11. Significance of maternal and paternal age interactions, quadratic parental age effects and environment by parental age interactions on female offspring and male offspring lifetime reproductive success in the Seychelles warbler. Significance was determined by a likelihood ratio test (LRT, P), df = 1, by adding the variable to the minimal model (Table 2). Significant fixed effects are highlighted in bold.

	**Female offspring LRS**		**Male offspring LRS**	
variables	LRT	P	LRT	P
age of mother^2	0.646	0.421	0.187	0.666
age of father^2	0.456	0.500	0.078	0.780
age of dominant male^2	0.480	0.488	0.072	0.788
territory quality*maternal age	0.626	0.429	0.082	0.775
territory quality*paternal age	**6.085**	**0.014**	0.009	0.926
territory quality*dominant male age	**4.698**	**0.030**	0.010	0.922
group size*maternal age	0.951	0.330	0.090	0.764
group size*paternal age	0.046	0.831	0.714	0.398
group size*dominant male age	1.773	0.183	0.086	0.770
helper*maternal age	0.530	0.467	0.612	0.434
helper*paternal age	0.013	0.911	0.772	0.380
helper*dominant male age	1.996	0.158	0.001	0.972
age of mother*age of father	0.397	0.529	0.207	0.649
age of mother*age of dominant male	1.503	0.220	0.011	0.917

Table S12. Generalised linear mixed model results investigating parental age effects (in years) on offspring annual reproductive success (measured as the number of offspring in the pedigree who survived to 1 year), in each sex separately, in the Seychelles warbler. Included are the estimated effects (estimate), standard errors (SEs), and significance of fixed effects based on a likelihood ratio test (LRT; P) where df=1. Significant fixed effects are highlighted in bold.

	**Female offspring ARS** n=2287 observations of 441 IDs				**Male offspring ARS** n=2341 observations of 466 IDs			
variables	estimate	SE	LRT	P	estimate	SE	LRT	P
fixed effects								
intercept	-1.912	0.118			-1.961	0.108		
age	4.983	0.399	-	-	4.870	0.356	-	-
age^2	**-4.935**	**0.435**	**211.530**	**<0.001**	**-4.043**	**0.331**	**213.390**	**<0.001**
lifespan	**0.392**	**0.134**	**8.407**	**0.004**	**0.350**	**0.132**	**7.017**	**0.008**
age of mother	-0.039	0.107	0.130	0.718	0.117	0.096	1.461	0.227
age of father	-0.015	0.119	0.016	0.900	0.128	0.114	1.256	0.263
age of dominant male	-0.139	0.117	1.416	0.234	0.161	0.113	2.032	0.154
hatch year	-0.036	0.207	0.030	0.863	-0.207	0.201	1.062	0.303
territory quality	-0.107	0.114	0.878	0.349	0.129	0.126	1.035	0.309
group size	0.090	0.111	0.634	0.426	-0.193	0.115	2.878	0.090
helper presence (y)	**-0.342**	**0.153**	**5.109**	**0.024**	0.010	0.147	0.005	0.944
sibling presence (y)	0.043	0.151	0.082	0.775	-0.058	0.136	0.183	0.669
random effects								
ID	0.070				0.066			
Year	0.153				0.120			

Table S13. Generalised linear mixed model results investigating between- versus within- maternal and paternal age effects on female offspring lifespan in the Seychelles warbler, using the within-subject centering method (van de Pol and Wright 2009). This model excludes the outlier seen in Figure S3, but model estimates are very similar with this outlier included (Table S4). Included are the estimated effects (estimate), standard errors (SEs), and significance of fixed effects based on a likelihood ratio test (LRT; P-value) where df=1. The model investigates within-parental age effects (deviation from the mean age of the parent) and between-parental age effects (mean age for each parent). Significant fixed effects are in bold.

	**Female offspring lifespan**			
	n=466			
variables	estimate	SE	LRT	P
fixed effects				
intercept	1.281	0.062		
between-maternal age	-0.104	0.090	1.309	0.253
within-maternal age	**-0.470**	**0.126**	**14.046**	**<0.001**
between-paternal age	0.012	0.110	0.011	0.916
within-paternal age	-0.162	0.134	1.460	0.227
between-dominant male age	0.111	0.094	1.383	0.240
within-dominant male age	0.240	0.147	2.680	0.102
hatch year	**-0.522**	**0.113**	**18.460**	**<0.001**
territory quality	0.021	0.081	0.067	0.796
group size	-0.100	0.077	1.704	0.192
helper presence (y)	-0.149	0.092	2.678	0.102
sibling presence (y)	-0.063	0.103	0.375	0.540
random effects				
maternal ID	0.086			
paternal ID	0.168			
dominant male ID	0.007			
hatch year	0.024			

Table S14. Generalised mixed model results investigating between- versus within- maternal and paternal age effects on female offspring lifetime reproductive success in the Seychelles warbler using the within-subject centering method (van de Pol and Wright 2009). This model excludes the outlier seen in Figure S4A, but model estimates are very similar with this outlier included (Table S9). Included are the estimated effects (estimate), standard errors (SEs), and significance of fixed effects based on a likelihood ratio test (LRT; P-value) where df=1. The model investigates within-parental age effects (deviation from the mean age of the parent) and between-parental age effects (mean age for each parent). Significant fixed effects are in bold.

	**Female offspring LRS**			
	n=440			
variables	estimate	SE	LRT	P
fixed effects				
intercept	0.509	0.131		
between-maternal age	-0.296	0.168	3.079	0.079
within-maternal age	**-0.847**	**0.298**	**7.695**	**0.006**
between-paternal age	0.044	0.178	0.061	0.806
within-paternal age	0.295	0.281	1.109	0.292
between-dominant male age	-0.141	0.171	0.689	0.407
within-dominant male age	0.027	0.303	0.008	0.928
hatch year	**-1.079**	**0.228**	**23.354**	**<0.001**
territory quality	-0.135	0.142	0.898	0.343
group size	0.003	0.154	<0.001	0.986
helper presence (y)	**-0.613**	**0.202**	**9.347**	**0.002**
sibling presence (y)	-0.110	0.190	0.338	0.561
random effects				
maternal ID	0.101			
paternal ID	0.094			
dominant male ID	0.049			

References

Brooks, M. E., K. Kristensen, K. J. van Benthem, A. Magnusson, C. W. Berg, A. Nielsen, H. J. Skaug, M. Maechler, and B. M. Bolker. 2017. glmmTMB Balances Speed and Flexibility Among Packages for Zero-inflated Generalized Linear Mixed Modeling. The R Journal 9:378–400.

Brouwer, L., D. S. Richardson, C. Eikenaar, and J. Komdeur. 2006. The role of group size and environmental factors on survival in a cooperatively breeding tropical passerine. Journal of Animal Ecology 75:1321–1329.

Brouwer, L., D. S. Richardson, and J. Komdeur. 2012. Helpers at the Nest Improve Late-Life Offspring Performance: Evidence from a Long-Term Study and a Cross-Foster Experiment. PLOS ONE 7:e33167.

Komdeur, J. 1992. Importance of habitat saturation and territory quality for evolution of cooperative breeding in the Seychelles warbler. Nature 358:493–495.

Raj Pant, S., J. Komdeur, T. Burke, H. L. Dugdale, and D. S. Richardson. 2019. Socio-ecological conditions and female infidelity in the Seychelles warbler. Behavioral Ecology 30:1254–1264.

van de Crommenacker, J., J. Komdeur, T. Burke, and D. S. Richardson. 2011. Spatio-temporal variation in territory quality and oxidative status: a natural experiment in the Seychelles warbler (Acrocephalus sechellensis). Journal of Animal Ecology 80:668–680.

van de Pol, M., and J. Wright. 2009. A simple method for distinguishing within- versus between-subject effects using mixed models. Animal Behaviour 77:753–758.
